# Immune priming in plants: from the onset to transgenerational maintenance

**DOI:** 10.1042/EBC20210082

**Published:** 2022-09-30

**Authors:** Agatha Cooper, Jurriaan Ton

**Affiliations:** School of Biosciences, PPS cluster, Institute for Sustainable Food, University of Sheffield, U.K.

**Keywords:** defence signalling, epigenetics, Induced resistance, plant Immunity, priming

## Abstract

Enhancing plant resistance against pests and diseases by priming plant immunity is an attractive concept for crop protection because it provides long-lasting broad-spectrum protection against pests and diseases. This review provides a selected overview of the latest advances in research on the molecular, biochemical and epigenetic drivers of plant immune priming. We review recent findings about the perception and signalling mechanisms controlling the onset of priming by the plant stress metabolite β-aminobutyric acid. In addition, we review the evidence for epigenetic regulation of long-term maintenance of priming and discuss how stress-induced reductions in DNA hypomethylation at transposable elements can prime defence genes. Finally, we examine how priming can be exploited in crop protection and articulate the opportunities and challenges of translating research results from the Arabidopsis model system to crops.

## Background

As sessile organisms, plants cannot run away from the variable and stressful conditions in their environment. Accordingly, they have evolved highly sophisticated and effective defence strategies to ensure survival and reproduction. Plant immunity is a research area that is of particular relevance for the development of sustainable agriculture. Current crop production systems are often based on genetically identical monocultures, which offer a suitable environment for pests and diseases to proliferate and inflict devastating yield losses. Increasing the efficiency of the plant immune system would negate the need to rely on unsustainable pesticides, which require substantial energy investment to produce and apply, and can have detrimental effects on the environment and human health.

### Plant innate immunity

The plant’s innate immune system operates according to a genetic blueprint and becomes active after detection of specific alarm signals. When a plant is under attack by a pathogen, pattern-recognition receptors (PRRs) respond to conserved microbe-associated molecular patterns (MAMPs) and/or damage-associated molecular patterns (DAMPs) that trigger downstream defence signalling cascades [1]. This process of threat detection and defence activation is called pattern-triggered immunity (PTI). The effectiveness of PTI is broad and allows resistance to become the rule rather than the exception. However, co-evolutionary pressures between plants and pathogens have resulted in a run-away evolutionary arms-race between immune-suppressing pathogen effectors and resistance genes, encoding nucleotide-binding leucine-rich repeater proteins (NB-LRRs) [2]. This extension of the plant innate immunity is known as effector-triggered immunity (ETI) and is highly effective against selected isolates of biotrophic pathogens but can rapidly become redundant upon emergence of new virulent strains that return the plant to the susceptible state.

### Induced resistance and priming

**Figure 1:**
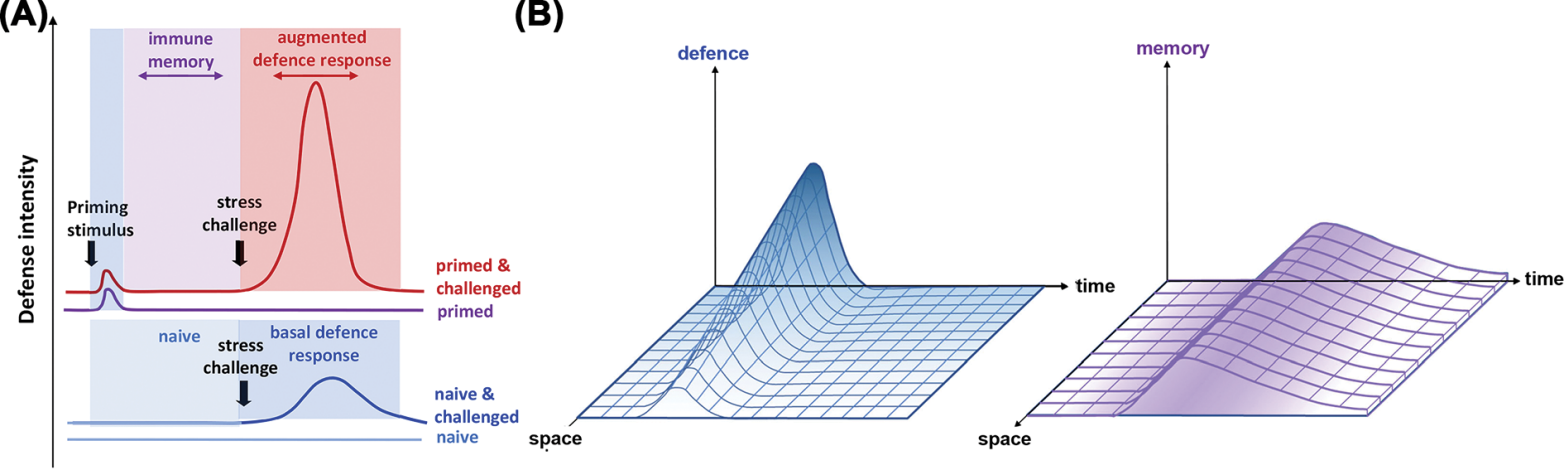
The priming model (**A**) Plants perceiving a priming stimulus transiently activate defence mechanisms after which they develop a longer lasting immunological memory, which allows them to mount a faster and/or stronger defence response upon secondary challenge compared to naïve plants. (**B**) The transient induction of defences at the site of attack sends signals throughout the plant but only lasts for a short period of time and is less pronounced at distal plant parts. The immune memory (purple) of the attack lasts much longer and can spread not only systemic plant parts but even to neighbouring plants via volatile signals and/or following generations via epigenetic mechanisms.

Plants can acquire increased levels of resistance after recovery from biotic stress. This ‘induced resistance’ (IR) is typically based on priming, which provides the plant with an enhanced defensive capacity that mediates a faster and/or stronger immune response upon future challenges by pests and diseases [3–5] (Figure 1A). Apart from priming that develops after recovery from biotic stress, priming can also be induced by non-pathogenic microbes [6] or chemical stimuli [7]. Examples of chemical priming stimuli are microbe-derived MAMPs such as chitin, or endogenous stress signalling compounds such as jasmonic acid (JA), salicylic acid (SA) or β-aminobutyric acid (BABA). In addition, there are xenobiotic chemicals such as benzothiadiazole (BTH) or (R)-β-homoserine (RBH), which partially mimic the activity of biological priming stimuli. While innate immunity is genetically hardwired into the DNA of the plant, priming is a form of phenotypic plasticity that is conceptually similar to acquired immunity in vertebrates, even though it relies on different mechanisms [4]. Primed plants are sensitized to ward off attackers and are capable of a faster and stronger induction of PTI-related defences than naïve plants that had been exposed to prior priming stimuli (Figure 1A). Due to the ecological costs of priming [8], priming is reversible, even though it can persist throughout the plant’s life cycle and, in some cases, be transmitted to following generations to offer protection against the same type of disease to which the parental plants had been exposed [9,10]. In addition to these temporal changes to the plant’s immune system, priming has a spatial dimension: it often develops in plant parts distal from the initial sites of attack through the action of long-distance (systemic) defence signals (Figure 1B,C). In some cases, priming can even be transmitted to other plants via volatile organic compounds (VOCs) [11–14].

IR by immune priming can vary depending on the eliciting signals, controlling signalling pathways and spectrum of effectiveness [7]. The three most intensely studied priming responses in plants are systemic acquired resistance (SAR), induced systemic resistance (ISR) and BABA-induced resistance (BABA-IR). SAR develops in response to local infection by pathogens and requires the stress hormone SA and the defence regulatory protein NPR1 [15]. SAR is mostly effective against biotrophic attackers [16] and is associated with a myriad of metabolic signals that prime SA/NPR1-dependent defences in distal plant parts against attack [17,18]. Induced systemic resistance (ISR) is activated in response to root colonization by beneficial non-pathogenic microbes, like mycorrhiza or rhizobacteria, and primes cell wall-based defences, JA and ethylene (ET)-dependent defence that are more effective against necrotrophic pathogens [6,16]. The response to the plant stress metabolite BABA has emerged as a popular model system to study the molecular signalling underpinning priming. This BABA-IR is based on priming of SA-dependent and -independent defences, providing broad-range protection against biotrophic pathogens, necrotrophic pathogens and even abiotic stresses [19–21].

Over recent years, there have been numerous reviews about IR and priming, each covering a range of molecular and biochemical mechanisms, such as increased accumulation of inactive defence signalling proteins (e.g. protein kinases and transcription factors) or glycosylated defence metabolites/hormones [4,5,22–24]. This review will therefore focus on a selection of recently emerged mechanisms of priming. We will first focus on the onset of priming by the plant endogenous stress metabolite BABA, and address how this compound is perceived by the plant and how it alters the defence signalling infrastructure of the cell to enable augmented defence induction upon pathogen challenge. Secondly, we provide a brief overview of epigenetic mechanisms by which priming can be maintained over expanding timescales. Finally, we assess the opportunities and challenges to translate this fundamental research into crop protection strategies.

## The onset of priming in the Arabidopsis-BABA model system

### The IBI1 receptor of BABA controls priming and plant stress via separate pathways

**Figure 2:**
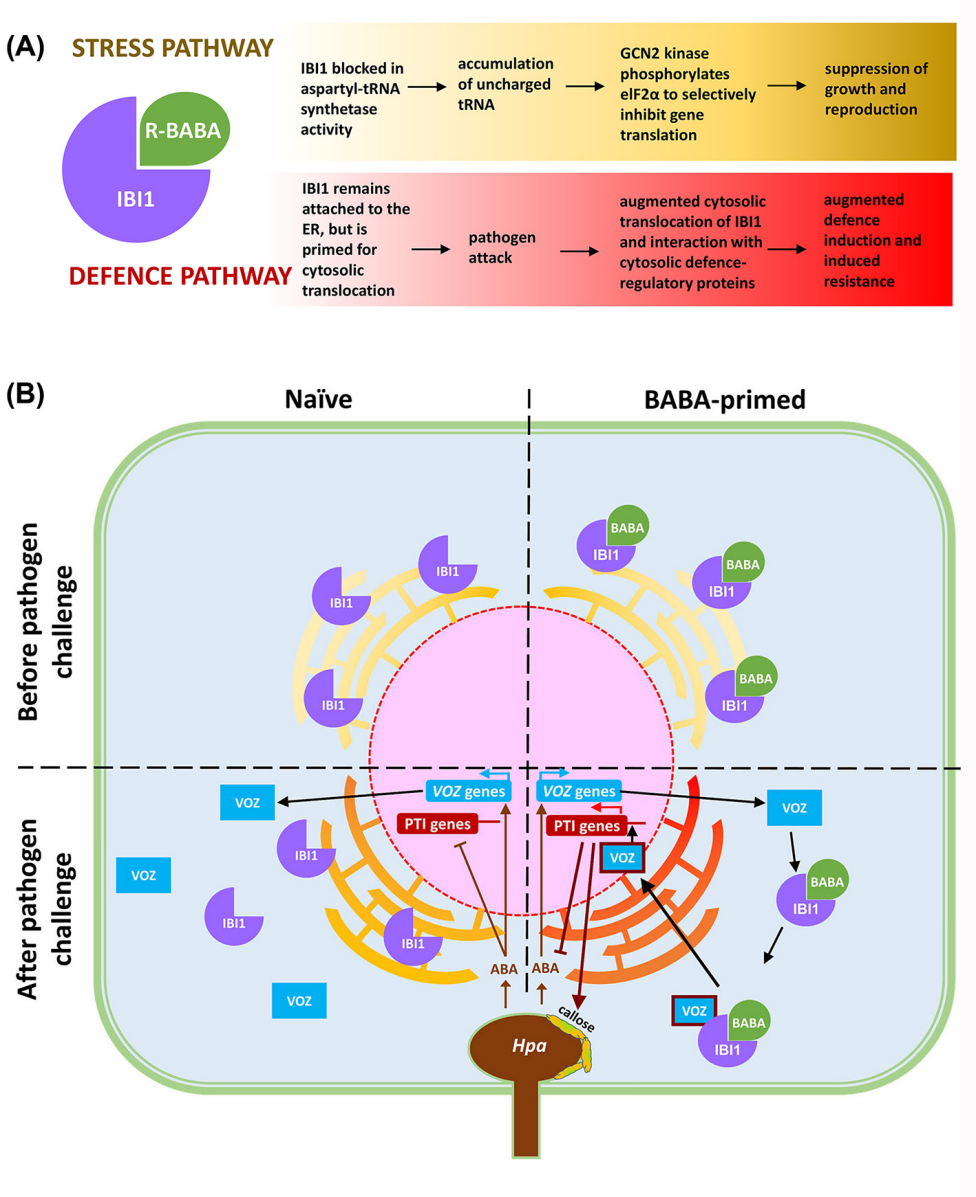
The IBI1 receptor of BABA controls BABA-induced stress (yellow) and BABA-induced resistance (red) via separate pathways (**A**) The R-enantiomer of BABA binds to the aspartyl tRNA synthetase IBI1 due to its structural similarity to L-aspartic acid. This disruptive binding prevents the charging of uncharged tRNA with L-aspartic acid and as a result, uncharged tRNA^Asp^ accumulates in the cell. Upon recognition of this uncharged tRNA by the GCN2 kinase, it phosphorylates the translation initiation factor eIF2α, which selectively inhibits the translation of genes involved in growth and reproduction, causing stress. At the same time, the binding of R-BABA to IBI1 primes the protein for augmented translocation from the endoplasmic reticulum (ER) to the cytosol, where it interacts with defence-regulatory proteins such as the VOZ1/2 transcription factors. (**B**) A cellular model of BABA induced resistance against the downy mildew pathogen *Hyaloperonospora arabidopsis* (*Hpa*; adapted from [32]). IBI1 is primarily located at the ER where it functions as an aspartyl tRNA synthetase. Binding of IBI1 to BABA after priming treatment loosens the anchorage of IBI1 to the ER, possibly through changes in ER membrane composition associated with ER stress. When attacked by *Hpa*, the cell accumulates abscisic acid (ABA) to suppress SA-dependent PTI. This virulence response simultaneously induces ABA-responsive *VOZ1* and *VOZ2* gene induction, resulting in an increased pool of VOZ1/2 transcription factors (TFs) in the cytosol. Simultaneously, the *Hpa*-induced translocation of IBI1 to the cytosol increases the chance for interaction between IBI1 and VOZ1/2 TFs, which activates VOZ1/2-dependent defence gene expression in the nucleus. Since IBI1 is primed to translocate to the cytosol, the interaction between IBI1 and VOZ1/2 occurs faster and stronger in BABA-treated plants after *Hpa* infection, resulting in augmented defence induction.

BABA is a non-proteinogenic β-amino acid that has been studied extensively for its resistance-inducing activities against viruses, pathogens, fungi and other microorganisms [25] as well as increasing tolerance to abiotic stresses like drought and salt stress [26]. Previously thought to be xenobiotic [21], it was recently found to be produced in low quantities upon exposure of plants to biotic and abiotic stresses [27]. BABA is not quickly metabolized in the cell, which partially explains why high doses of BABA can severely affect plant growth and fertility [25]. More recently, it was shown that BABA-IR and BABA-induced stress are controlled by different signalling pathways (Figure 2A) [28]. The active R-enantiomer of BABA binds to the L-aspartic acid binding pocket of the aspartyl-tRNA synthetase IBI1, which acts as a cellular receptor of BABA [28,29]. This interaction inhibits the aspartyl-tRNA synthetase activity by IBI1, resulting in cellular accumulation of its upstream substrates: L-aspartic acid and uncharged tRNA^asp^ [28]. The build-up of uncharged tRNA is commonly associated with amino acid limitation in eukaryotic cells. In plants, this cellular stress activates a salvation pathway that is under control by the tRNA-sensing GCN2 kinase, which phosphorylates the eukaryotic translation initiation factor eIF2α that in turn selectively inhibits the translation of genes involved in growth and reproduction [30]. Interestingly, the collagen-suppressing drug halofuginone (HF) was recently found to trigger similar responses in mammalian cells by inhibition of glutamyl-prolyl-tRNA: in addition to activating a GCN2-dependent amino acid starvation response, the drug modulated the immune activity by cytokine-stimulated fibroblast-like synoviocytes [31]. It thus appears as if aminoacyl-tRNA synthetases are promising targets to manipulate immune responses in both plants and humans.

### Downstream signalling components in the IBI1-dependent IR pathway

Recently, Schwarzenbacher et al. [32] identified a new signalling step in the BABA-IR pathway, which acts immediately downstream of the perception of BABA by IBI1. It was previously shown that the IBI1 receptor is localized at the endoplasmic reticulum (ER) and that BABA primes pathogen-induced translocation of the receptor to the cytoplasm, where it was hypothesized that it interacts with defence regulatory defence signalling proteins (Figure 2B) [28]. Based on yeast-two-hybrid profiling, the Vascular Plant One Zinc Finger 1 (VOZ1) and VOZ2 were identified as interactors of IBI1, which was confirmed by *in planta* bimolecular fluorescence complementation analysis. VOZ1/2 are transcription factors that are predominantly localized in the cytosol but small fractions of this cytosolic pool migrate into the nucleus to activate downstream genes [33]. The function of VOZ1/2 in BABA-IR was validated by the finding that the *voz1 voz2* double mutant is impaired in BABA-induced priming for callose-associated cell wall defences. Previously, the plant hormone abscisic acid (ABA) has been implicated in BABA-induced priming of callose-associated cell wall defences [20,34], but it always remained difficult to reconcile this finding with the fact that ABA suppresses SA-dependent PTI [35,36], which is exploited by virulent pathogens to mediate effector-triggered susceptibility [37]. Schwarzenbacher et al. [32] provided an answer to this apparent paradox by demonstrating that VOZ1/VOZ2 are transcriptionally induced by ABA during downy mildew infection. They proposed that the BABA-induced priming for increased IBI1 translocation [28] allows it to interact more readily with ABA-induced VOZ1/2 during pathogen infection, resulting in increased VOZ1/2 activity in the nucleus to mediate augmented induction of early-acting PTI genes involved in cell wall defence.

### The endoplasmic reticulum: a regulator of IBI1-dependent priming?

The default localization of IBI1 to the endoplasmic reticulum (ER) points to a regulatory role of the ER in BABA-induced priming, particularly as the ER is emerging as an important regulator of innate immunity in eukaryotic cells [38]. Indeed, ER stress and the associated unfolded protein response (UPR) have been shown to control PTI [39]. In that regard, it is plausible that PTI-related ER stress acts as a trigger of the defence-related translocation of IBI1 from the ER to the cytoplasm, where it interacts with VOZ transcription factors to trigger PTI genes. It is also noteworthy that Schwarzenbacher et al. [32] identified the ER-localized fatty acid hydroxylase 2 (FAH2) as an interactor of IBI1. This ER-localized enzyme has previously been shown to mediate 2-hydroxylation of palmitic acid (PA) [40], which *in planta* mostly occurs at the PA chain of glycosyl-ceramides [41]. Interestingly, transport of 2-hydroxy-sphingolipids from the ER to the plasma membrane (PM) has recently been implicated in early-acting immune responses of rice to chitin [42]. Given the immune-regulatory role of ER stress [39], it is tempting to speculate that BABA-induced changes in the interaction between IBI1 and FAH2 alter ER membrane composition, which in turn primes the translocation of IBI1 from the ER to the cytosol to mediate augmented VOZ-dependent defence during pathogen attack.

## Long-term maintenance of priming

**Figure 3:**
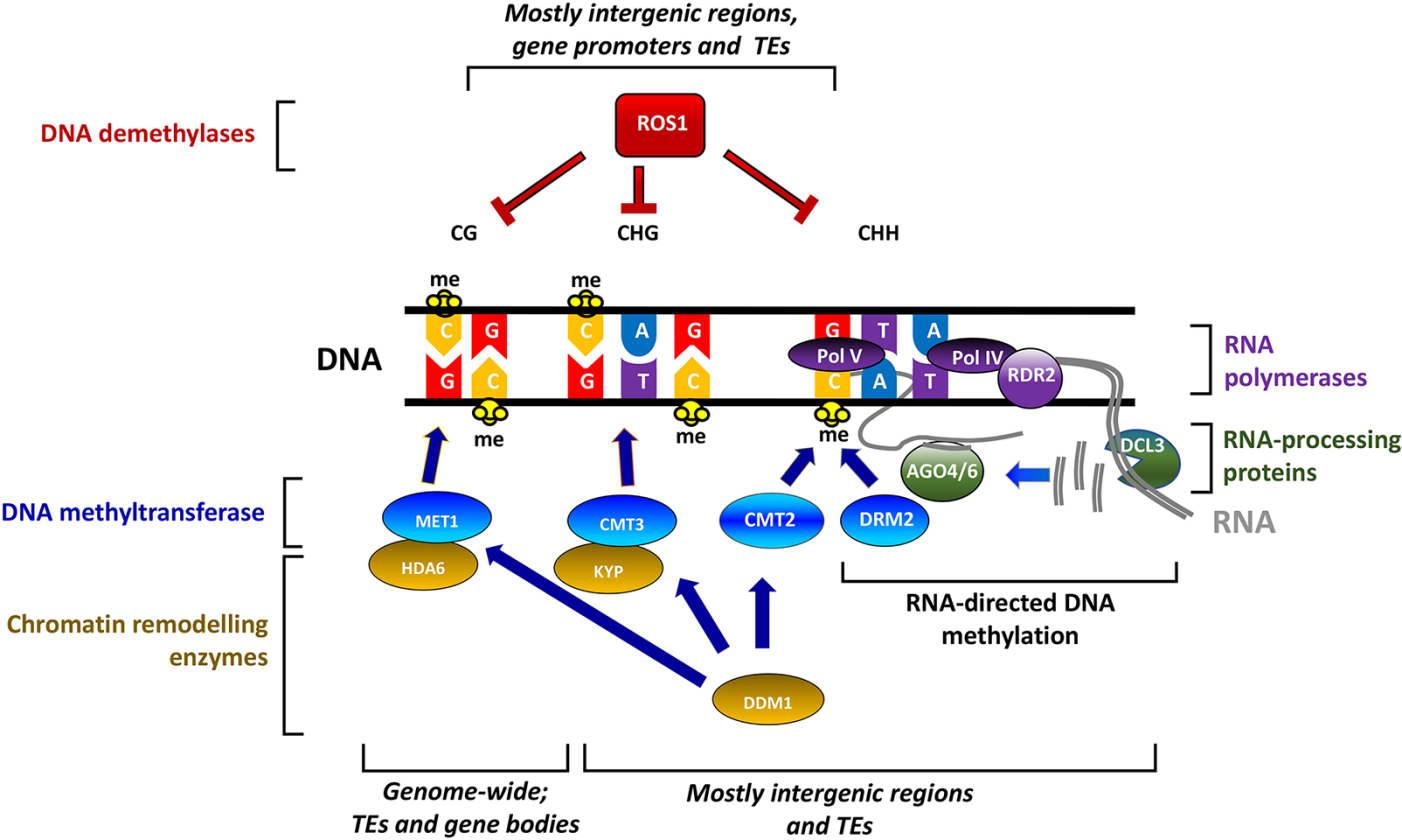
DNA methylation homeostasis in plants DNA methylation predominantly occurs at the 5-carbon of cytosine and typically signals for tightly packed chromatin (heterochromatin) to prevent transcription by RNA polymerase II. DNA methylation in plants occurs at three different sequence contexts: CG, CHG and CHH where H stands for any nucleotide aside from guanine [4]. While DNA methylation is most prevalent at transposable elements (TEs), it occurs throughout the plant genome, including gene bodies [47]. In Arabidopsis, the induction and establishment of cytosine methylation is mediated RNA-directed DNA methylation (RdDM), which involves RNA polymerases IV and V, small interfering RNAs (siRNAs), AGO (Argonaute) proteins and the DNA methyltransferase DRM2, which methylates DNA at CHH context [48,49]. Not shown are variations to the RdDM pathway that initiate CHH methylation (often referred to as non-canonical RdDM, which involves RNA pol II, AGO1 and RDR6 [50]). Once established, methylation at CG sequence context is maintained by MET1 (Methyltransferase 1), while the methyltransferases CMT2 and CMT3 (Chromomethylase 2/3) maintain methylation at CHH and CHG contexts, respectively [51]. These DNA methyl transferases often interact directly or indirectly with chromatin remodelling enzymes to ensure tightly packed heterochromatin. Demethylation is executed by four demethylases, DME (Demeter), DML2 (Demeter-Like 2), DML3 (Demeter-Like 3) and ROS1 (Repressor Of Silencing 1), of which the latter is expressed in vegetative tissues [52]. The antagonistic activities between the DNA demethylases and the methylation pathways determines the level of DNA methylation at transposable elements [53].

The first systematic study of IR in tobacco by Ross [43] in 1961 reported long-lasting protection that lasted up to at least several weeks. This durability of IR implies that stress-exposed tissues transmit a resistance-inducing state into newly formed cell lines, which remains stable over iterative cell divisions. Most IR research over subsequent decades focused on the spatial distribution of systemic priming relatively shortly after localized induction treatment and largely ignored the long-term maintenance of IR. In 2012 however, three independent research groups reported that pathogen- or herbivore-treated Arabidopsis can prime their progeny for enhanced phytohormone-dependent defences, resulting in transgenerational IR [9,44,45]. Supported by an earlier report that chemical priming of pathogen-inducible defence genes in Arabidopsis is associated with post-translational modifications of histone H3 proteins in the corresponding gene promoters [46], these independent reports pointed to an important function of epigenetic mechanisms in the long-term maintenance of priming. Over the following decade, more evidence emerged for the involvement of epigenetic mechanisms in priming maintenance [23]. The next section of our review focuses more specifically on the mechanisms by which stress-induced changes in DNA methylation control priming of defence gene expression. Figure 3 provides a simplified scheme of the main mechanisms controlling DNA methylation homeostasis.

### The role of DNA demethylation in priming

Various studies have shown that genetic mutations affecting DNA methylation have a profound impact on disease resistance. Dowan et al. [54] demonstrated that *met1* and *ddc* mutants, which are both severely DNA hypo-methylated, displayed strongly increased levels of resistance against *Pseudomonas syringae* pv. *tomato* (*Pst*), suggesting that DNA methylation suppresses resistance against biotrophic pathogens. Yu et al. [55] reached a similar conclusion by demonstrating that the DNA hypermethylated *ros1* mutant of Arabidopsis shows increased susceptibility to *Pst*. More recently, this latter group furthermore demonstrated that ROS1 *cis*-regulates the defence genes *RMG1* and *RLP43* by erasing DNA methylation at regulatory sequences in the promoters of these genes, which explains the compromised resistance of the *ros1* mutant to *P. syringae* [56]. The link between DNA demethylation and transgenerational immune priming was made by López Sánchez et al. [57], who demonstrated that *ros1* is impaired in transgenerational IR by *Pst*, while this mutation does affect short-term within-generation IR. Using epigenetic recombinant inbred lines (epiRILs) from a cross between Col-0 wild-type plants and the TE hypomethylated *ddm1-2* mutant, Furci et al. [58] demonstrated that heritable DNA hypomethylation at selected TE-rich regions causes genome-wide priming of defence genes and high levels of disease resistance. Together, these studies provided causal evidence for a role of TE methylation in transgenerational priming. It is commonly assumed that hypomethylated TEs can induce and/or prime the expression of genes controlling PTI. The following section reviews various mechanisms by which PTI genes can be influenced in this manner.

### Mechanisms by which transposable elements prime defence genes: *cis* versus *trans*

**Figure 4:**
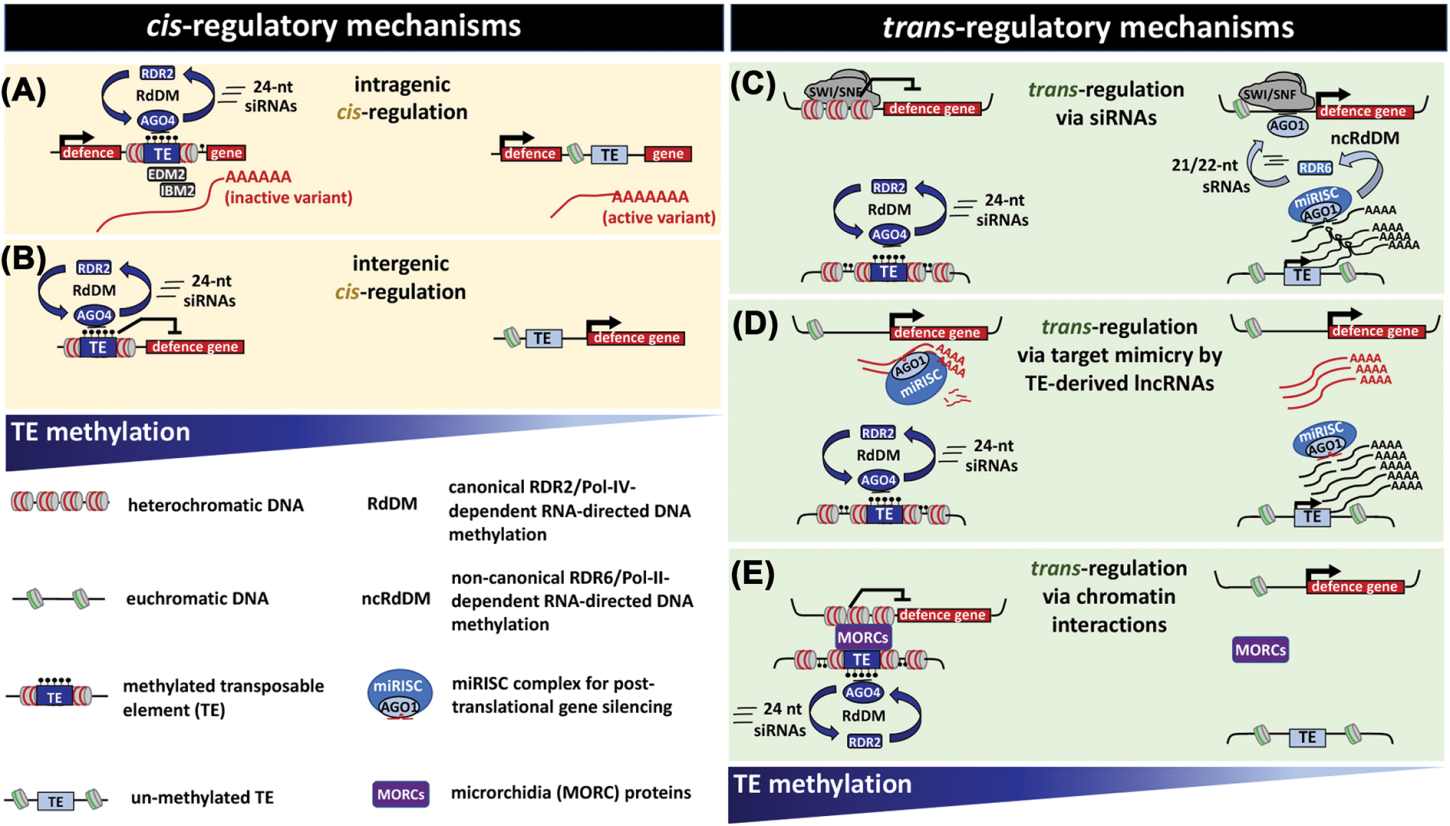
Model of *cis*- and *trans*-regulation of defence gene priming by hypomethylated TEs *Cis*-regulation (left) occurs when an intronic TE controls alternative splicing or polyadenylation of the defence gene (**A**), or when the DNA methylation status of a nearby TE controls the chromatin density and transcriptional responsiveness of a defence gene (**B**). *Trans*-regulation (right) can be based on a variety of mechanisms. Post-transcriptional silencing of transcribed TEs by the RNA-induced silencing complex (RISC) can induce accumulation of 21/22-nt sRNAs via RDR6-dependent RNA-directed DNA methylation. This can induce nuclear activity of argonaute1 (AGO1), which stimulates defence gene expression through interaction with the SWI/SNF chromatin remodelling complex in a sRNA-dependent manner (**C**; [61]). Alternatively, transcription of hypomethylated TEs can increase accumulation of non-coding RNAs (lncRNAs) that scavenge defence-repressing microRNAs (miRNAs) through target mimicry (**D**; [63,64]). Finally, salicylic acid-binding microrchidia (MORC) proteins can mediate higher-order heterochromatic interactions in the genome [65,66], and so regulate the chromatin structure and expression of distal defence genes (**E**; [58]).

Despite emerging evidence for a role of TE methylation in the long-term maintenance of immune priming, there remains debate as to how stress-induced hypomethylation of TEs controls defence genes in primed plants. *Cis-*regulation, whereby the defence gene is controlled by a nearby TE, is the most straightforward explanation. In this scenario, stress-induced hypomethylation of the TE changes the chromatin status of genes, which in turn modifies the transcriptional capacity and splicing of the associated defence gene (Figure 4) [23,59]. However, this model of *cis*-regulation may not be the only mechanism by which hypomethylated TEs control defence genes. Cambiagno et al. [60] reported that *Pst* transiently induces the expression of pericentromeric TEs, which results in the accumulation of RdDM-related sRNAs that map to both TEs and defence genes, including genes encoding pattern recognition receptor (PRR). Interestingly, while RdDM was effective in re-silencing the TEs, the complementary defence genes at distal genomic locations remained active, suggesting *trans*-regulation by TE-derived sRNAs. Liu et al. [61] demonstrated that stressed plants generate AGO1-associated siRNAs, which *trans*-activate distal defence genes through interaction with the SWI/SNF chromatin remodelling complex and recruitment of stalled RNA pol-II, a mechanism that had previously been linked to priming of stress-inducible genes [62]. In addition, it is conceivable that hypomethylated TEs are transcribed into long non-coding RNAs (lncRNAs), which act as target mimics of defence-repressing miRNAs (Figure 4). Finally, Furci et al. [58] demonstrated that none of the hypomethylated TEs within the resistance-enhancing quantitative trait loci (epiQTL) from the Col-0 × *ddm1-2* epiRIL population were associated with nearby defence genes. By mining publicly available Hi-C data from Col-0 and *ddm1-2* plants, they furthermore showed that many hypo-methylated TEs in the epiQTL form DDM1-dependent long-range heterochromatic interactions with distal defence genes, suggesting another possible mechanism by which hypo-methylated TEs *trans*-prime defence genes (Figure 4). Clearly, more research is needed to consolidate these hypotheses. Previous research has provided the foundation by demonstrating transgenerational priming in plants and the role of DNA demethylation therein, but future research should focus on the spatiotemporal scale in which this epigenetic memory is established, maintained and translated into an augmented immune response during pathogen attack.

## Implications for crop protection

With future climate change and a projected increase in the human population impending, new and more sustainable crop protection strategies are needed. This can only be achieved by translating basic knowledge about the functioning of the plant immune system into new management and breeding strategies that increase durable plant resistance.

Exploiting priming-inducing chemicals (priming agents) as ‘plant vaccines’ is an attractive concept but also needs careful consideration. A major hurdle against wide-spread adoption of chemical priming is their variability between different pathogen-interactions and their undesirable side effects on plant growth [7]. For instance, despite the broad-spectrum effectiveness of BABA-IR, its adoption as a crop protection agent is hampered by the fact that it represses plant growth at higher concentrations [8,25,29]. However, the discovery of the IBI1 receptor and the accompanying finding that increased expression of *IBI1* not only enhances BABA-IR efficiency but also increases plant tolerance to BABA-induced stress [28] and provides major opportunities to combine BABA with targeted crop breeding to maximize the cost-benefit balance of BABA-IR [28]. Alternatively, Buswell et al. [29] identified a chemical BABA analogue, RBH, which primes the plant for different defence pathways and is less toxic than BABA, generating opportunities to combine sub-toxic doses of BABA with RBH. Furthermore, a primed immune state can be engineered through genetically modified (GM) approaches. For instance, the recent discovery that defence genes are regulated at the translational level [67,68] has been exploited to engineer constitutively primed crop varieties without major costs to plant growth. Xu et al. [69] cloned the pathogen-responsive upstream open reading frames (uORFs) of the *TBF* gene to drive augmented translational induction of the *NPR1* gene in rice, resulting in broad-spectrum disease resistance without the costs incurred by constitutive transcription of *NPR1*. These chemical and transgenic approaches illustrate that it is possible to uncouple the protective benefits of immune priming from the associated costs on plant growth. Integration of these strategies with other resistance breeding strategies, like pyramiding of resistance (R) genes [70], would not only improve sustainable crop protection but also protect R genes against co-evolutionary pressures by pathogens.

**Figure 5:**
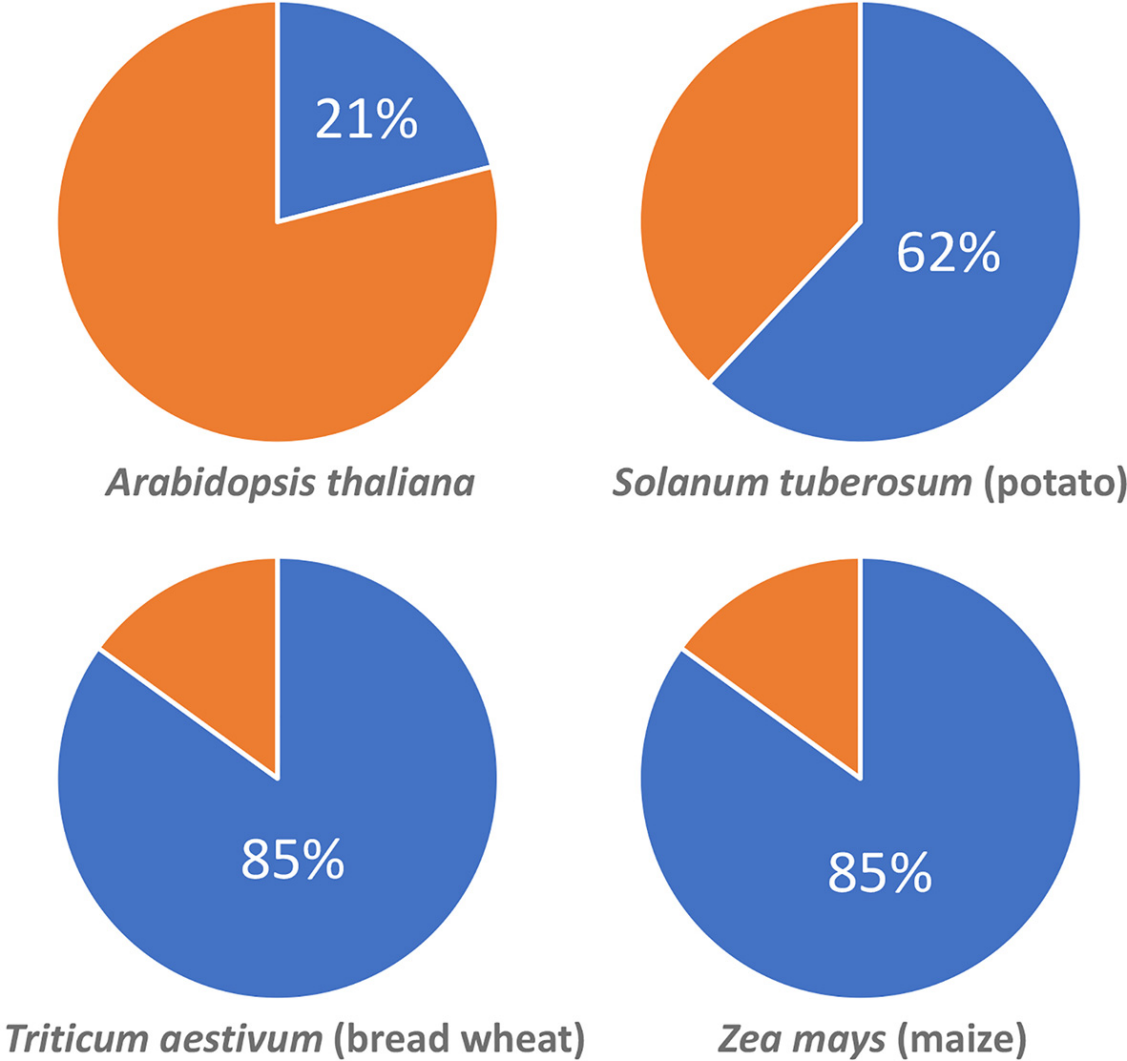
Estimated percentages of genomic sequence covered by transposable elements (TEs; blue) in Arabidopsis and three different crop species Data gathered from TAIR 10.1 for Arabidopsis, Lee and Kim [76] for maize, Wicker et al. [77] for bread wheat and The Potato Genome Consortium [78] for potato.

The latest insights about epigenetic regulation of priming also offer opportunities for translation into durable crop protection. For instance, seeds from defence-elicited parental plants could be harvested and exploited to offer better disease protection [71–73]. However, IR by transgenerational priming is typically weaker and less consistent than within-generational priming responses. Moreover, López Sánchez et al. [10] showed that there are ecological costs associated with transgenerational IR, mostly arising from increased susceptibility to other stresses than those triggering the IR response. Arguably a more efficient way to exploit epigenetically controlled IR is by directly manipulating the epigenome and selecting for epi-genotypes that are primed for multiple plant defence pathways without compromising effects on plant growth or resistance to other stresses. In Arabidopsis, Furci et al. [58] provided proof-of-concept by demonstrating that selected epiRILs from the Col-0 × *ddm1-2* cross are more resistant to both biotrophic and necrotrophic pathogens without associated reductions in plant growth. Accordingly, it is tempting to assume that similar approaches in crops can generate epigenetically primed varieties with high levels of disease protection without costs on plant growth. However, generating epigenetically altered crop varieties has proven difficult because crop genomes have much higher numbers of TEs (Figure 5) and are, therefore, more sensitive to genome-wide reductions in DNA methylation than Arabidopsis resulting in lethal or sterile phenotypes [74]. Accordingly, more adjustable methods are required to introduce DNA hypomethylation in crops, which may prevent lethality/sterility from over-stimulation, whilst still ensuring sufficient impact to mediate epi-IR. The development of gene constructs that enable spatiotemporal ectopic control of DNA demethylase genes, as well as the recent advances in the exploitation of CRISPR-dCas constructs for epigenomic editing [75], offer realistic opportunities to achieve this goal.

## Summary

- Priming is a form of immunological memory in plants that increases the responsiveness of the immune system against pests and diseases. Although the effectiveness, duration and specificity of the resulting induced resistance (IR) response depends on the eliciting stimulus, priming is typically expressed throughout the plant (i.e. systemically) and is long-lasting.
- The response of Arabidopsis to the plant stress metabolite β-aminobutyric acid (BABA) has emerged as a model system to study the signalling pathways mediating the onset of priming. Recent studies of this model system have revealed key regulatory roles of abscisic acid (ABA) and the endoplasmic reticulum (ER).
- Long-term maintenance of priming has an epigenetic basis and involves regulation by DNA hypomethylation at transposable mechanisms.
- Exploitation of the chemical priming agent BABA requires careful consideration of the associated trade-offs on plant growth. In Arabidopsis, the balance between disease protection and phytotoxicity can be optimized by genetic manipulation of the BABA receptor gene *IBI1* and/or combinations of sub-toxic doses of other chemical priming agents.
- Genetic engineering of pathogen-responsive upstream open reading frames (uORFs) to drive augmented translational induction of the defence-inducing genes can generate constitutively primed crop varieties that are not compromised in growth.
- Stress-induced epigenetic priming can be mimicked and enhanced by reducing the level of DNA methylation at transposable elements in the plant genome. To prevent major non-target effects on crop growth and fertility, exploitation of this epigenetic immune priming requires new tools to precisely control and target the level of epigenomic variation.

## Open Access

Open access for this article was enabled by the participation of University of Sheffield in an all-inclusive *Read & Publish* agreement with Portland Press and the Biochemical Society under a transformative agreement with JISC.

## Abbreviations

ABA: abscisic acid
AGO1: argonaute1
BABA: β-aminobutyric acid
BTH: benzothiadiazole
CMT2/3: chromomethylase 2/3
DME: demeter
DML2/3: demeter-like 2/3
DAMP: damage-associated molecular pattern
epiRIL: epigenetic recombinant inbred line
ER: endoplasmic reticulum
ET: ethylene
ETI: effector-triggered immunity
FAH2: fatty acid hydroxylase 2
GM: genetically modified
HF: halofuginone
IR: induced resistance
ISR: induced systemic resistance
JA: jasmonic acid
lncRNA: long non-coding RNA
MAMP: microbe-associated molecular pattern
MET1: methyltransferase 1
miRNA: microRNA
MORC: microrchidia
NB-LRR: nucleotide-binding leucine-rich repeater protein
PA: palmitic acid
PM: plasma membrane
PRR: pattern recognition receptor
PTI: pattern-triggered immunity
R: resistance
RBH: (R)-β-homoserine
RdDM: RNA-directed DNA methylation
RISC: RNA-induced silencing complex
ROS1: repressor of silencing 1
SA: salicylic acid
SAR: systemic acquired resistance
siRNA: small interfering RNA
TE: transposable element
uORF: upstream open reading frame
UPR: unfolded protein response
VOC: volatile organic compound
VOZ1/2: vascular plant one zinc finger 1/2

## References

[1] Muthamilarasan M. and Prasad M. (2013) Plant innate immunity: an updated insight into defense mechanism. J. Biosci. 38, 433–449 10.1007/s12038-013-9302-2 23660678

[2] Hurley B., Subramaniam R., Guttman D.S. and Desveaux D. (2014) Proteomics of effector-triggered immunity (ETI) in plants. Virulence 5, 752–760 10.4161/viru.36329 25513776 PMC4189881

[3] Pastor V., Luna E., Mauch-Mani B., Ton J. and Flors V. (2013) Primed plants do not forget. Environ. Exp. Bot. 94, 46–56 10.1016/j.envexpbot.2012.02.013

[4] Wilkinson S.W., Magerøy M.H., López Sánchez A., Smith L.M., Furci L., Cotton T.E.A. et al. (2019) Surviving in a hostile world: Plant strategies to resist pests and diseases. Annu. Rev. Phytopathol. 57, 505–529 10.1146/annurev-phyto-082718-095959 31470772

[5] Mauch-Mani B., Baccelli I., Luna E. and Flors V. (2017) Defense priming: an adaptive part of induced resistance. Annu. Rev. Plant Biol. 68, 485–512 10.1146/annurev-arplant-042916-041132 28226238

[6] Pieterse C.M.J., Zamioudis C., Berendsen R.L., Weller D.M., Van Wees S.C.M. and Bakker P.A.H.M. (2014) Induced systemic resistance by beneficial microbes. Annu. Rev. Phytopathol. 52, 347–375 10.1146/annurev-phyto-082712-102340 24906124

[7] Yassin M., Ton J., Rolfe S.A., Valentine T.A., Cromey M., Holden N. et al. (2021) The rise, fall and resurrection of chemical‐induced resistance agents. Pest Manag. Sci. 77, 3900–3909 10.1002/ps.6370 33729685

[8] van Hulten M., Pelser M., van Loon L.C., Pieterse C.M.J. and Ton J. (2006) Costs and benefits of priming for defense in Arabidopsis. Proc. Natl. Acad. Sci. U.S.A. 103, 5602–5607 10.1073/pnas.0510213103 16565218 PMC1459400

[9] Luna E., Bruce T.J.A., Roberts M.R., Flors V. and Ton J. (2012) Next-generation systemic acquired resistance. Plant Physiol. 158, 844–853 10.1104/pp.111.187468 22147520 PMC3271772

[10] López Sánchez A., Pascual-Pardo D., Furci L., Roberts M.R. and Ton J. (2021) Costs and benefits of transgenerational induced resistance in Arabidopsis. Front. Plant Sci. 12, 644999 10.3389/fpls.2021.644999 33719325 PMC7952753

[11] Engelberth J., Alborn H.T., Schmelz E.A. and Tumlinson J.H. (2004) Airborne signals prime plants against insect herbivore attack. Proc. Natl. Acad. Sci. U.S.A. 101, 1781–1785 10.1073/pnas.0308037100 14749516 PMC341853

[12] Erb M., Veyrat N., Robert C.A.M., Xu H., Frey M., Ton J. et al. (2015) Indole is an essential herbivore-induced volatile priming signal in maize. Nat. Commun. 6, 6273 10.1038/ncomms7273 PMC4339915 25683900

[13] Ton J., D'Alessandro M., Jourdie V., Jakab G., Karlen D., Held M. et al. (2007) Priming by airborne signals boosts direct and indirect resistance in maize. Plant J. 49, 16–26 10.1111/j.1365-313X.2006.02935.x 17144894

[14] Ye K., Li H., Ding Y., Shi Y., Song C., Gong Z. et al. (2019) BRASSINOSTEROID-INSENSITIVE2 negatively regulates the stability of transcription factor ICE1 in response to cold stress in Arabidopsis. Plant Cell 31, 2682–2696 10.1105/tpc.19.00058 31409630 PMC6881119

[15] Spoel S.H. and Dong X. (2012) How do plants achieve immunity? Defence without specialized immune cells Nat. Rev. Immunol. 12, 89–100 10.1038/nri3141 22273771

[16] Ton J., Van Pelt J.A., Van Loon L.C. and Pieterse C.M.J. (2002) Differential effectiveness of salicylate-dependent and jasmonate/ethylene-dependent induced resistance in Arabidopsis. Mol. Plant. Microbe. Interact. 15, 27–34 10.1094/MPMI.2002.15.1.27 11858171

[17] Kachroo A. and Kachroo P. (2020) Mobile signals in systemic acquired resistance. Curr. Opin. Plant Biol. 58, 41–47 10.1016/j.pbi.2020.10.004 33202317

[18] Zeier J. (2021) Metabolic regulation of systemic acquired resistance. Curr. Opin. Plant Biol. 62, 102050 10.1016/j.pbi.2021.102050 34058598

[19] Zimmerli L., Jakab G., Métraux J.-P. and Mauch-Mani B. (2000) Potentiation of pathogen-specific defense mechanisms in Arabidopsis by β-aminobutyric acid. Proc. Natl. Acad. Sci. U.S.A. 97, 12920–12925 10.1073/pnas.230416897 11058166 PMC18865

[20] Ton J. and Mauch-Mani B. (2004) β-amino-butyric acid-induced resistance against necrotrophic pathogens is based on ABA-dependent priming for callose. Plant J. 38, 119–130 10.1111/j.1365-313X.2004.02028.x 15053765

[21] Cohen Y., Vaknin M. and Mauch-Mani B. (2016) BABA-induced resistance: milestones along a 55-year journey. Phytoparasitica 44, 513–538 10.1007/s12600-016-0546-x

[22] Balmer A., Pastor V., Gamir J., Flors V. and Mauch-Mani B. (2015) The ‘prime-ome’: towards a holistic approach to priming. Trends Plant Sci. 20, 443–452 10.1016/j.tplants.2015.04.002 25921921

[23] Hannan Parker A., Wilkinson S.W. and Ton J. (2022) Epigenetics: a catalyst of plant immunity against pathogens. New Phytol. 233, 66–83 10.1111/nph.17699 34455592

[24] Conrath U., Beckers G.J.M., Langenbach C.J.G. and Jaskiewicz M.R. (2015) Priming for enhanced defense. Annu. Rev. Phytopathol. 53, 97–119 10.1146/annurev-phyto-080614-120132 26070330

[25] Jakab G., Cottier V., Toquin V., Rigoli G., Zimmerli L., Métraux J.-P. et al. (2001) β-aminobutyric acid-induced resistance in plants. Eur. J. Plant Pathol. 107, 29–37 10.1023/A:1008730721037

[26] Jisha K.C. and Puthur J.T. (2015) Seed priming with BABA (β-amino butyric acid): a cost-effective method of abiotic stress tolerance in Vigna radiata (L.) Wilczek. Protoplasma 253, 277–289 10.1007/s00709-015-0804-7 25837010

[27] Thevenet D., Pastor V., Baccelli I., Balmer A., Vallat A., Neier R. et al. (2017) The priming molecule β-aminobutyric acid is naturally present in plants and is induced by stress. New Phytol. 213, 552–559 10.1111/nph.14298 27782340

[28] Luna E., van Hulten M., Zhang Y., Berkowitz O., López A., Pétriacq P. et al. (2014) Plant perception of β-aminobutyric acid is mediated by an aspartyl-tRNA synthetase. Nat. Chem. Biol. 10, 450–456 10.1038/nchembio.1520 24776930 PMC4028204

[29] Buswell W., Schwarzenbacher R.E., Luna E., Sellwood M., Chen B., Flors V. et al. (2018) Chemical priming of immunity without costs to plant growth. New Phytol. 218, 1205–1216 10.1111/nph.15062 29465773

[30] Li M.W., AuYeung W.K. and Lam H.M. (2013) The GCN2 homologue in Arabidopsis thaliana interacts with uncharged tRNA and uses Arabidopsis eIF2α molecules as direct substrates. Plant Biol. (Stuttg.) 15, 13–18 10.1111/j.1438-8677.2012.00606.x 22672016

[31] Kim Y., Sundrud M.S., Zhou C., Edenius M., Zocco D., Powers K. et al. (2020) Aminoacyl-tRNA synthetase inhibition activates a pathway that branches from the canonical amino acid response in mammalian cells. Proc. Natl. Acad. Sci. U.S.A. 117, 8900–8911 10.1073/pnas.1913788117 32253314 PMC7183223

[32] Schwarzenbacher R.E., Wardell G., Stassen J., Guest E., Zhang P., Luna E. et al. (2020) The IBI1 receptor of β-aminobutyric acid interacts with VOZ transcription factors to regulate abscisic acid signaling and callose-associated defense. Mol. Plant 13, 1455–1469 10.1016/j.molp.2020.07.010 32717347 PMC7550849

[33] Yasui Y., Mukougawa K., Uemoto M., Yokofuji A., Suzuri R., Nishitani A. et al. (2012) The phytochrome-interacting vascular plant one-zinc finger1 and VOZ2 redundantly regulate flowering in Arabidopsis. Plant Cell. 24, 3248–3263 10.1105/tpc.112.101915 22904146 PMC3462629

[34] Flors V., Ton J., van Doorn R., Jakab G., García-Agustín P. and Mauch-Mani B. (2008) Interplay between JA, SA and ABA signalling during basal and induced resistance against Pseudomonas syringae and Alternaria brassicicola. Plant J. 54, 81–92 10.1111/j.1365-313X.2007.03397.x 18088307

[35] Yasuda M., Ishikawa A., Jikumaru Y., Seki M., Umezawa T., Asami T. et al. (2008) Antagonistic interaction between systemic acquired resistance and the abscisic acid–mediated abiotic stress response in Arabidopsis. Plant Cell. 20, 1678–1692 10.1105/tpc.107.054296 18586869 PMC2483369

[36] Fan J., Hill L., Crooks C., Doerner P. and Lamb C. (2009) Abscisic acid has a key role in modulating diverse plant-pathogen interactions. Plant Physiol. 150, 1750–1761 10.1104/pp.109.137943 19571312 PMC2719142

[37] Asai S., Rallapalli G., Piquerez S.J.M., Caillaud M.-C., Furzer O.J., Ishaque N. et al. (2014) Expression profiling during Arabidopsis/downy mildew interaction reveals a highly-expressed effector that attenuates responses to salicylic acid. PLoS Pathog. 10, e1004443 10.1371/journal.ppat.1004443 25329884 PMC4199768

[38] Kørner C.J., Du X., Vollmer M.E. and Pajerowska-Mukhtar K.M. (2015) Endoplasmic reticulum stress signaling in plant immunity - at the crossroad of life and death. Int. J. Mol. Sci. 16, 26582–26598 10.3390/ijms161125964 26556351 PMC4661823

[39] Verchot J. and Pajerowska-Mukhtar K.M. (2021) UPR signaling at the nexus of plant viral, bacterial, and fungal defenses. Curr. Opin. Virol. 47, 9–17 10.1016/j.coviro.2020.11.001 33360330

[40] Nagano M., Takahara K., Fujimoto M., Tsutsumi N., Uchimiya H. and Kawai-Yamada M. (2012) Arabidopsis sphingolipid fatty acid 2-hydroxylases (AtFAH1 and AtFAH2) are functionally differentiated in fatty acid 2-hydroxylation and stress responses. Plant Physiol. 159, 1138–1148 10.1104/pp.112.199547 22635113 PMC3387700

[41] König S., Feussner K., Schwarz M., Kaever A., Iven T., Landesfeind M. et al. (2012) Arabidopsis mutants of sphingolipid fatty acid α-hydroxylases accumulate ceramides and salicylates. New Phytol. 196, 1086–1097 10.1111/j.1469-8137.2012.04351.x 23025549

[42] Nagano M., Ishikawa T., Fujiwara M., Fukao Y., Kawano Y., Kawai-Yamada M. et al. (2016) Plasma membrane microdomains are essential for Rac1-RbohB/H-mediated immunity in rice. Plant Cell. 28, 1966–1983 10.1105/tpc.16.00201 27465023 PMC5006704

[43] Ross A.F. (1961) Systemic acquired resistance induced by localized virus infections in plants. Virology 14, 340–358 10.1016/0042-6822(61)90319-1 13743578

[44] Slaughter A., Daniel X., Flors V., Luna E., Hohn B. and Mauch-Mani B. (2012) Descendants of primed Arabidopsis plants exhibit resistance to biotic stress. Plant Physiol. 158, 835–843 10.1104/pp.111.191593 22209872 PMC3271771

[45] Rasmann S., De Vos M., Casteel C.L., Donglan T., Halitschke R., Sun J.Y. et al. (2012) Herbivory in the previous generation primes plants for enhanced insect resistance. Plant Physiol. 158, 854–863 10.1104/pp.111.187831 22209873 PMC3271773

[46] Jaskiewicz M., Conrath U. and Peterhänsel C. (2011) Chromatin modification acts as a memory for systemic acquired resistance in the plant stress response. EMBO Rep. 12, 50–55 10.1038/embor.2010.186 21132017 PMC3024125

[47] Bewick A.J. and Schmitz R.J. (2017) Gene body DNA methylation in plants. Curr. Opin. Plant Biol. 36, 103–110 10.1016/j.pbi.2016.12.007 28258985 PMC5413422

[48] Zhang H., Lang Z. and Zhu J.-K. (2018) Dynamics and function of DNA methylation in plants. Nat. Rev. Mol. Cell Biol. 19, 489–506 10.1038/s41580-018-0016-z 29784956

[49] McCue A.D., Panda K., Nuthikattu S., Choudury S.G., Thomas E.N. and Slotkin R.K. (2015) ARGONAUTE 6 bridges transposable element mRNA-derived siRNAs to the establishment of DNA methylation. EMBO J. 34, 20–35 10.15252/embj.201489499 25388951 PMC4291478

[50] Cuerda-Gil D. and Slotkin R. (2016) Non-canonical RNA-directed DNA methylation. Nat. Plants 2, 16163 10.1038/nplants.2016.163 27808230

[51] Liang W., Li J., Sun L., Liu Y., Lan Z. and Qian W. (2022) Deciphering the synergistic and redundant roles of CG and non-CG DNA methylation in plant development and transposable element silencing. New Phytol. 233, 722–737 10.1111/nph.17804 34655488 PMC9298111

[52] Deleris A., Halter T. and Navarro L. (2016) DNA methylation and demethylation in plant immunity. Annu. Rev. Phytopathol. 54, 579–603 10.1146/annurev-phyto-080615-100308 27491436

[53] Tang K., Lang Z., Zhang H. and Zhu J.-K. (2016) The DNA demethylase ROS1 targets genomic regions with distinct chromatin modifications. Nat. Plants 2, 16169 10.1038/nplants.2016.169 27797352 PMC5123759

[54] Dowen R.H., Pelizzola M., Schmitz R.J., Lister R., Dowen J.M. and Nery J.R. (2012) Widespread dynamic DNA methylation in response to biotic stress. Proc. Natl. Acad. Sci. U.S.A. 109, 2183–2191 10.1073/pnas.1209329109 PMC3420206 22733782

[55] Yu A., Lepère G., Jay F., Wang J., Bapaume L., Wang Y. et al. (2013) Dynamics and biological relevance of DNA demethylation in Arabidopsis antibacterial defense. Proc. Natl. Acad. Sci. U.S.A. 110, 2389–2394 10.1073/pnas.1211757110 23335630 PMC3568381

[56] Halter T., Wang J., Amesefe D., Lastrucci E., Charvin M., Singla Rastogi M. et al. (2021) The Arabidopsis active demethylase ROS1 cis-regulates defence genes by erasing DNA methylation at promoter-regulatory regions. eLife 10, e62994 10.7554/eLife.62994 33470193 PMC7880685

[57] López Sánchez A., Stassen J.H.M., Furci L., Smith L.M. and Ton J. (2016) The role of DNA (de)methylation in immune responsiveness of Arabidopsis. Plant J. 88, 361–374 10.1111/tpj.13252 27341062 PMC5132069

[58] Furci L., Jain R., Stassen J., Berkowitz O., Whelan J., Roquis D. et al. (2019) Identification and characterisation of hypomethylated DNA loci controlling quantitative resistance in Arabidopsis. eLife 8, e40655 10.7554/eLife.40655 30608232 PMC6342528

[59] Lai Y., Lu X.M., Daron J., Pan S., Wang J., Wang W. et al. (2020) The Arabidopsis PHD-finger protein EDM2 has multiple roles in balancing NLR immune receptor gene expression. PLos Genet. 16, e1008993 10.1371/journal.pgen.1008993 32925902 PMC7529245

[60] Cambiagno D.A., Nota F., Zavallo D., Rius S., Casati P., Asurmendi S. et al. (2018) Immune receptor genes and pericentromeric transposons as targets of common epigenetic regulatory elements. Plant J. 96, 1178–1190 10.1111/tpj.14098 30238536

[61] Liu C., Xin Y., Xu L., Cai Z., Xue Y., Liu Y. et al. (2018) Arabidopsis ARGONAUTE 1 binds chromatin to promote gene transcription in response to hormones and stresses. Dev. Cell 44, 348–361 10.1016/j.devcel.2017.12.002 29290588

[62] Ding Y., Fromm M. and Avramova Z. (2012) Multiple exposures to drought ‘train’ transcriptional responses in Arabidopsis. Nat. Commun. 3, 740 10.1038/ncomms1732 22415831

[63] Wu H.-J., Wang Z.-M., Wang M. and Wang X.-J. (2013) Widespread long noncoding RNAs as endogenous target mimics for microRNAs in plants. Plant Physiol. 161, 1875–1884 10.1104/pp.113.215962 23429259 PMC3613462

[64] Canto-Pastor A., Santos B.A.M.C., Valli A.A., Summers W., Schornack S. and Baulcombe D.C. (2019) Enhanced resistance to bacterial and oomycete pathogens by short tandem target mimic RNAs in tomato. Proc. Natl. Acad. Sci. U.S.A. 116, 2755–2760 10.1073/pnas.1814380116 30679269 PMC6377479

[65] Moissiard G., Cokus S.J., Cary J., Feng S., Billi A.C., Stroud H. et al. (2012) MORC family ATPases required for heterochromatin condensation and gene silencing. Science 336, 1448–1451 10.1126/science.1221472 22555433 PMC3376212

[66] Manohar M., Choi H.W., Manosalva P., Austin C.A., Peters J.E. and Klessig D.F. (2017) Plant and human MORC proteins have DNA-modifying activities similar to Type II topoisomerases, but require one or more additional factors for full activity. Mol. Plant-Microbe Interact. 30, 87–100 10.1094/MPMI-10-16-0208-R 27992291

[67] Pajerowska-Mukhtar K.M., Wang W., Tada Y., Oka N., Tucker C.L., Fonseca J.P. et al. (2012) The HSF-like transcription factor TBF1 is a major molecular switch for plant growth-to-defense transition. Curr. Biol. 22, 103–122 10.1016/j.cub.2011.12.015 22244999 PMC3298764

[68] Xu G., Greene G., Yoo H., Liu L., Marqués J., Motley J. et al. (2017) Global translational reprogramming is a fundamental layer of immune regulation in plants. Nature 545, 487–490 10.1038/nature22371 28514447 PMC5485861

[69] Xu G., Yuan M., Ai C., Liu L., Zhuang E., Karapetyan S. et al. (2017) uORF-mediated translation allows engineered plant disease resistance without fitness costs. Nature 545, 491–494 10.1038/nature22372 28514448 PMC5532539

[70] Mundt C.C. (2018) Pyramiding for resistance durability: theory and practice. Phytopathology 108, 792–802 10.1094/PHYTO-12-17-0426-RVW 29648947

[71] Walters D.R. and Paterson L. (2012) Parents lend a helping hand to their offspring in plant defence. Biol. Lett. 8, 871–873 10.1098/rsbl.2012.0416 22696290 PMC3440994

[72] Ramírez-Carrasco R., Martínez-Aguilar K. and Alvarez-Venegas R. (2017) Transgenerational defense priming for crop protection against plant pathogens: a hypothesis. Front. Plant Sci. 8, 696 10.3389/fpls.2017.00696 28523009 PMC5415615

[73] Akköprü A. (2020) Potential using of transgenerational resistance against common bacterial blight in Phaseolus vulgaris. Crop. Prot. 127, 104967 10.1016/j.cropro.2019.104967

[74] Rudd J. (2017) Plant epigenetics: an untapped molecular resource for crop improvement. Technol. Networks Genomics Res. [Internet] Available from https://www.technologynetworks.com/genomics/articles/plant-epigenetics-an-untapped-molecular-resource-for-crop-improvement-290477 (accessed 14 July 2017)

[75] Gjaltema R.A.F. and Rots M.G. (2020) Advances of epigenetic editing. Curr. Opin. Chem. Biol. 57, 75–81 10.1016/j.cbpa.2020.04.020 32619853

[76] Lee S.-I. and Kim N.-S. (2014) Transposable elements and genome size variations in plants. Genomics Inform. 12, 87–97 10.5808/GI.2014.12.3.87 25317107 PMC4196380

[77] Wicker T., Gundlach H., Spannagl M., Uauy C., Borrill P., Ramírez-González R.H. et al. (2018) Impact of transposable elements on genome structure and evolution in bread wheat. Genome Biol. 19, 103 10.1186/s13059-018-1479-0 30115100 PMC6097303

[78] The Potato Genome Consortium (2011) Genome sequence and analysis of the tuber crop potato. Nature 475, 189–195 10.1038/nature10158 21743474

